# Adaptive chromatin remodeling and transcriptional changes of the functional kinome in tumor cells in response to targeted kinase inhibition

**DOI:** 10.1016/j.jbc.2021.101525

**Published:** 2021-12-24

**Authors:** Michael P. East, Gary L. Johnson

**Affiliations:** Department of Pharmacology and Lineberger Comprehensive Cancer Center, University of North Carolina at Chapel Hill, Chapel Hill, North Carolina, USA

**Keywords:** kinome, kinase inhibitor, adaptive resistance, chromatin remodeling, enhancer formation, transcription, ABL1, abelson tyrosine-protein kinase 1, AKT, RAC-alpha serine/threonine-protein kinase, BCR, breakpoint cluster region protein, BET, bromo- and extra-terminal, BRAF, murine sarcoma viral oncogene homolog B, BRD4, bromodomain containing 4, CBP/p300, CREB binding protein and p300 are histone acetyltransferases, CDC42BPA/B/G, CDC42 binding protein kinase alpha/beta/gamma, CDH1, cadherin 1, CDK1/19, cyclin dependent kinase 1/19, CDKL5, cyclin dependent kinase-like 5, CML, chronic mylogenous leukemia, c-MYC, MYC proto-oncogene, COL6A2, collagen type VI alpha 2 chain, DDR1, epithelial discoidin domain-containing receptor 1, EGFR, epidermal growth factor receptor, EpCAM, epithelial cell adhesion molecule, EPHB4, ephrin type-B receptor 4, ERK1/2, mitogen-activated protein kinase 1/2, EZH2, enhancer of zeste 2 polycomb repressive complex 2 subunit, FAIRE, formaldehyde assisted isolation of regulatory elements, FDA, food and drug administration, FES, feline sarcoma oncogene, FGFR2, fibroblast growth factor receptor 2, FOXA1, forkhead box A1, FRK, fyn related kinase, GRHL2, grainyhead-like transcription factor 2, HDAC, histone deacetylase, HER2/3, human epidermal growth factor receptor 2/3, IGF-1, insulin-like growth factor 1, IGF1R, insulin-like growth factor 1 receptor, JAK1, janus kinase 1, KDM5A/6A/6B, lysine demethylase 5A/6A/6B, KRAS, Kirsten rat sarcoma viral oncogene homolog, KRT5, keratin 5, LYN, LYN proto-oncogene, MED1, mediator complex subunit 1, MET, hepatocyte growth factor receptor, MIB/MS, multiplexed inhibitor beads coupled with mass spectrometry, NSCLC, non-small cell lung carcinoma, PDGFRβ, platelet derived growth factor receptor beta, PDX, patient-derived xenograft, PI3K, phosphoinositide 3-kinase, P-TEFb, positive transcription elongation factor, PTK7, protein tyrosine kinase 7, RAS, rat sarcoma viral homolog, RTK, receptor tyrosine kinase, SHP2/PTPN11, protein tyrosine phosphatase nonreceptor type 11, SMARCD3/BAF50c, SWI/SNF-related matrix-associated actin-dependent regulator of chromatin subfamily D member 3, SWI/SNF, switch/sucrose nonfermentable, TK, nonreceptor tyrosine kinase, TK1, thymidine kinase 1, TNBC, triple negative breast cancer, VEGFR2, vascular endothelial growth factor receptor 2

## Abstract

Pharmacological inhibition of protein kinases induces adaptive reprogramming of tumor cell regulatory networks by altering expression of genes that regulate signaling, including protein kinases. Adaptive responses are dependent on transcriptional changes resulting from remodeling of enhancer and promoter landscapes. Enhancer and promoter remodeling in response to targeted kinase inhibition is controlled by changes in open chromatin state and by activity of specific transcription factors, such as c-MYC. This review focuses on the dynamic plasticity of protein kinase expression of the tumor cell kinome and the resulting adaptive resistance to targeted kinase inhibition. Plasticity of the functional kinome has been shown in patient window trials where triple-negative and human epidermal growth factor receptor 2–positive breast cancer patient tumors were characterized by RNAseq after biopsies before and after 1 week of therapy. The expressed kinome changed dramatically during drug treatment, and these changes in kinase expression were shown in cell lines and xenografts in mice to be correlated with adaptive tumor cell drug resistance. The dynamic transcriptional nature of the kinome also differs for inhibitors targeting different kinase signaling pathways (*e.g.*, BRAF-MEK-ERK *versus* PI3K-AKT) that are commonly activated in cancers. Heterogeneity arising from differences in gene regulation and mutations represents a challenge to therapeutic durability and prevention of clinical drug resistance with drug-tolerant tumor cell populations developing and persisting through treatment. We conclude that understanding the heterogeneity of kinase expression at baseline and in response to therapy is imperative for development of combinations and timing intervals of therapies making interventions durable.

Protein kinases function in signaling networks controlling most cellular functions and their dysfunction contributes to many human diseases, most notably cancer. Most human cell lines express ∼350–400 protein kinases that are integrated into canonical signaling networks. In different cancers, kinases are frequently mutated or amplified making them excellent targets for inhibition to inhibit growth and/or induce apoptosis of the tumor cell (1). The first targeted kinase inhibitor, gleevec (imatinib also known as STI571), was approved in 2001 to treat chronic myelogenous leukemia (CML). CML is generally caused by the fusion of the ABL1 gene (Abelson tyrosine kinase 1) and BCR gene (Breakpoint Cluster Region gene) resulting in the constitutive activation of the ABL1 tyrosine kinase (2). Imatinib is a competitive inhibitor binding to the ATP active site of ABL1 that revolutionized the treatment of CML, but ∼30% of patients fail to achieve lasting results due to development of Imatinib resistance (3, 4).

A second early success in the development of kinase inhibitors was targeting HER2+ (ERBB2) breast cancer. HER2, human epidermal growth factor receptor 2, is a tyrosine kinase and is amplified in ∼25% of breast cancers. HER2 inhibition drastically improves patient outcome, but efficacy can be short-lived, and resistance frequently develops. Small-molecule inhibitors of the HER2 and EGFR kinases include lapatinib, tucatinib, and the irreversible inhibitor neratinib (5). Monoclonal antibodies such as trastuzumab and pertuzumab are also effective in HER2+ breast cancer and have different mechanisms of action. Trastuzumab targets the HER2 extracellular domain and in combination with chemotherapy in early breast cancer decreases death by nearly 40%. A major mechanism of trastuzumab involves the recruitment of immune cells to the HER2+ tumor (6). Upregulation of HER3, the preferred heterodimerization partner of HER2 results in HER2-HER3 heterodimers that drive PI3K-AKT activation (7). Pertuzumab blocks HER2-HER3 dimerization and significantly improves outcomes when added to trastuzumab-based therapy (8). HER2+ tumors that respond to the different HER2-directed therapies often develop acquired resistance resulting in disease progression (9, 10). The adaptive response also results in upregulation of additional genes including kinases that overcome the initial HER2 inhibition (10, 11, 12, 13).

Currently, there are 66 kinase inhibitors approved by the Food and Drug Administration (FDA) with many more in development (14). Drugging the human kinome with targeted inhibitors has been hampered by the development of drug resistance, and many if not most kinase inhibitors as single agents fail to produce durable responses in the clinic as monotherapies. While Darwinian selection of cells harboring acquired kinase mutations may be overcome by development of new inhibitors effective against the mutant kinase (15, 16, 17, 18), resistance mediated by adaptive kinome reprogramming remains a significant clinical concern. In this review, we address the mechanisms of adaptive reprogramming of the kinome in response to targeted kinase inhibition and how this must be overcome by combination and timing of treatments to make kinase inhibitor therapies durable.

## A chromatin-mediated drug tolerant state

Tumor cells can acquire genetic mutations that render the targeted protein insensitive to a drug to induce acquired drug resistance (19, 20, 21, 22). In contrast, cells may rapidly adapt to a targeted therapy, for example, by adaptive kinome reprogramming (adaptive resistance). An example of the adaptive resistance mechanism was described for a “drug tolerant state” in the non-small-cell lung cancer (NSCLC) PC9 cell line (22). PC9 cells express an activated mutant form of EGFR having deletion of Glu746–Ala750 in exon 19 that drives cellular proliferation and a dependency for survival of PC9 cells. The cells are extremely sensitive to the EGFR inhibitors gefitinib and erlotinib with growth arrest and loss of viability (22). However, a small number of EGFR inhibitor-treated PC9 cells “persist” and escape cell death during EGFR inhibitor treatment, and with several days of continuous inhibitor treatment, drug-tolerant persister cells begin to propagate. There was no acquired mutation or amplification of the EGFR or expression of the receptor tyrosine kinase MET in the drug-tolerant PC9 cells that is often seen in drug-resistant patient NSCLC (23). Notably, the drug-tolerant state was reversible, as cells reacquired drug sensitivity after drug withdrawal and could emerge *de novo* from clonally isolated, drug-sensitive cells. The persister cells were driven by IGF-1 receptor tyrosine kinase signaling and upregulation of the histone demethylase KDM5A, which regulates function of histones in chromatin by regulating their methylation of specific lysine residues (22). IGF-1 receptor inhibition or knockdown of KDM5A was sufficient to restore drug sensitivity, demonstrating a chromatin-dependent regulation of the drug-tolerant state. Selective inhibitors of KDM5A were unavailable, but inhibitors of histone deacetylases (HDACs) killed cells in the drug-tolerant state but not parental cells. These data are consistent with a mechanism for acute drug tolerance that is mediated by chromatin remodeling, where chromatin accessibility is heavily regulated by the posttranslational modification of histones by enzymes such as KDM5A and different HDACs to control gene transcription and is comprehensively reviewed elsewhere (24).

A second study in EGFR-mutant NSCLC showed similar epigenetic reprogramming in the drug-tolerant persister cells using histone mass spectrometry assays (25). Drug-tolerant cells showed higher global H3K27me3 and H3K9me3 marks combined with a decrease in multiple H3Kacetylation (H3Kac) marks. H3K27me3 and H3K9me3 are associated with heterochromatin and a decrease in expression of nearby genes, whereas increased H3Kac is associated with a more open chromatin state and an increase in transcription of nearby genes. An siRNA screen of chromatin regulators also revealed dependencies on the histone methyltransferase EZH2, H3K9 methylation-dependent chromatin regulators, and several HDACs. In a study in triple-negative breast cancer (TNBC), targeted inhibition of kinases in the two primary proliferative kinase cascades-MEK1/2-ERK1/2 pathway (MEK-ERK) or PI3K-AKT pathway resulted in a reversible drug-tolerant state with profound effects on chromatin accessibility of specific transcription factor motifs (26). MEK or PI3K inhibition showed upregulation in activity of BRD4, which binds acetylated histones to promote gene expression. A BRD4 bromo- and extra-terminal domain (BET) inhibitor, JQ1, suppressed the drug-tolerant state and synergized with MEK and PI3K inhibitors. JQ1 also inhibited the changes in open chromatin state, bringing open motifs back to near DMSO control states. Similar epigenetic reprogramming was observed in glioblastoma in response to targeted kinase inhibitors with epigenetic reprogramming of H3K27ac and H3K27me3 marks accompanied by a dependency on the histone demethylase KDM6A/B for drug tolerance (27).

## Adaptive kinome reprogramming

Changes in expression of protein kinases represent adaptive kinome reprogramming, which has been shown to regulate the onset of resistance to targeted kinase inhibitors. Using Multiplexed Inhibitor Beads coupled with Mass spectrometry (MIB/MS) to capture and identify functional protein kinases, Duncan *et al.* showed that treatment of TNBC cell lines with selumetinib, an allosteric inhibitor of MEK in the MEK1/2-ERK1/2 MAPK pathway, resulted in the expression and activation of multiple protein kinases including several receptor tyrosine kinases (RTKs) (28). The expression of RTKs, including DDR1, PDGFRβ, and VEGFR2, resulted in overcoming MEK-ERK inhibition allowing reactivation of tumor cell growth. Zawistowski *et al.* showed that MEK inhibition by trametinib induced a transcriptional response in multiple TNBC cell lines (29). Other studies have defined adaptive kinome reprogramming in different tumor types (*e.g.*, renal cell carcinoma, ovarian cancer, leukemia, glioblastoma lymphoma) and with inhibitors targeting kinases in pathways different from the MEK-ERK pathway such as targeting inhibition of the PI3K-AKT pathway (11, 30, 31, 32, 33, 34, 35, 36, 37, 38, 39, 40).

## Transcriptional response in patient tumors to targeted kinase inhibition

**Figure 1:**
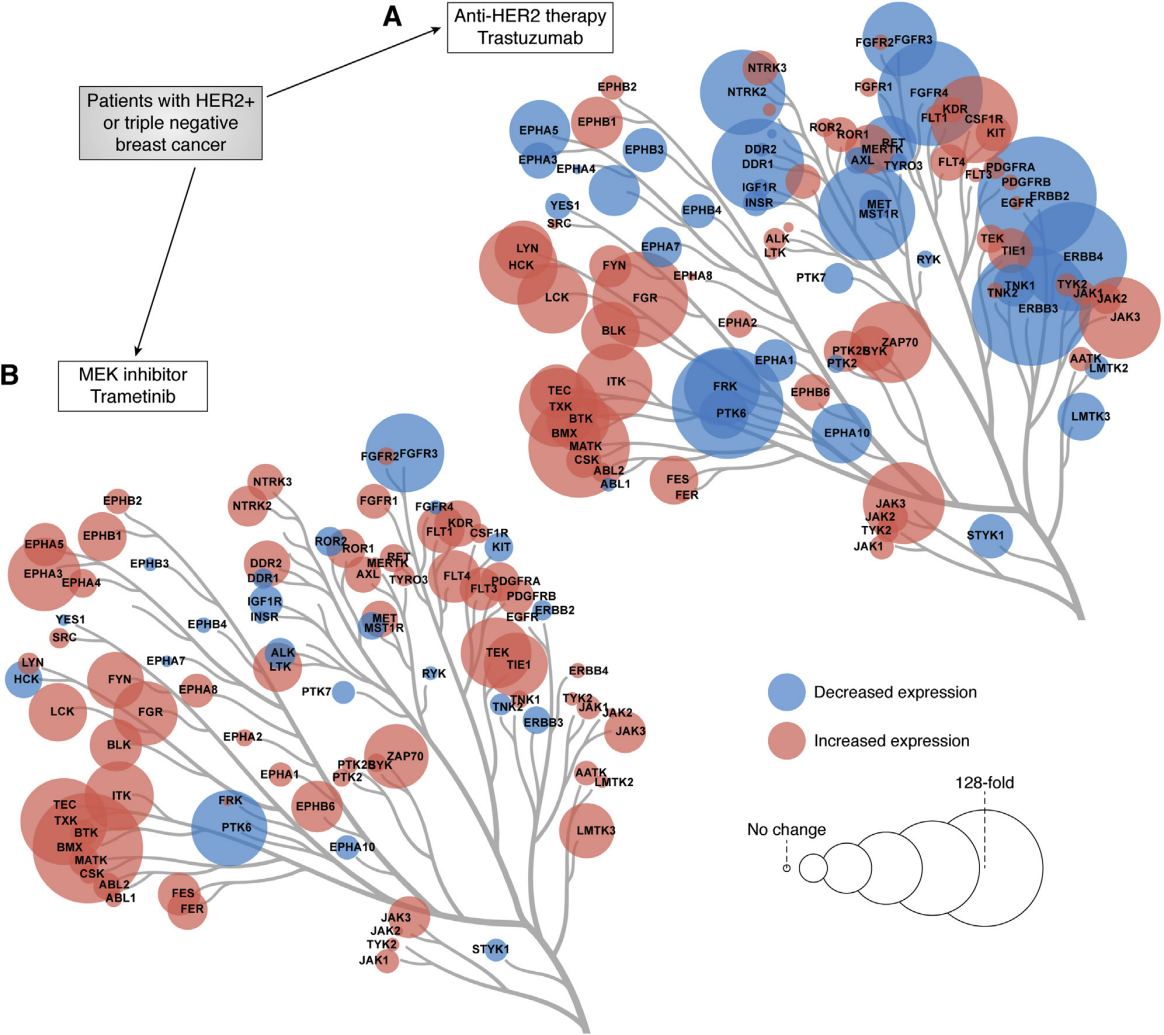
**Tyrosine kinase dynamics in response to targeted kinase inhibition in two independent window-of-opportunity clinical trials.** Patients with previously untreated HER2+ or triple negative breast cancer were treated with anti-HER2 therapy trastuzumab (*A*) or MEK inhibitor trametinib (*B*), respectively, for 7 days. Tumor biopsies were taken prior to treatment and on day 7 for transcriptional profiling by RNA-seq. Phylogenetic trees of tyrosine kinases (TK) are shown from representative patients. *Red* and *blue circles* indicate up- and downregulation of TK expression levels in response to drug treatment. *Circle* size corresponds to the magnitude of expression level changes with the *largest circles* indicating a 128-fold change in expression in response to drug treatment. Figures reproduced from published data (29, 47). Phylogenetic trees were generated with Coral (87).

Transcriptional and kinome reprogramming is observed in patients receiving targeted kinase inhibitors. In a window-of-opportunity trial, women having TNBC with no prior treatment received trametinib for 7 continuous days (29) (Fig. 1). Biopsies were taken before and after the 7-day treatment for five basal-like and one claudin-low tumors and analyzed by RNAseq. A strong transcriptomic response was seen in the tumors with up to 22% of the transcriptome being significantly up- or downregulated, functionally resulting in a phenotypic state change of the tumors. Multiple RTKs were upregulated in trametinib-treated tumors relative to pretreatment biopsies. Proteomic analysis demonstrated the RTKs were functionally expressed binding to immobilized kinase inhibitors measured by MIB/MS. The five basal-like tumors showed a heterogeneous adaptive kinome reprogramming with different RTKs being upregulated after trametinib treatment in the patient tumors. The claudin-low tumor showed unique adaptive kinome reprogramming compared with the basal-like tumors. The findings were similar to what has been characterized for the adaptive kinome reprogramming response to trametinib in basal-like and claudin-low TNBC cell lines and mouse xenografts (28, 29). Using DEseq2 for differential expression analysis showed six protein kinases were transcriptionally upregulated in both patient basal-like breast cancers and three basal-like cell lines: DDR1, HER2, FRK, CDC42BPG, CDK19, and CDKL5 (Fig. 1). DDR1, HER2, and FRK are tyrosine kinases that would have the ability to activate both the MEK-ERK and PI3K-AKT pathways to overcome trametinib inhibition of the MEK-ERK pathway. CDC42BPG, CDKL5, and CDK19 are serine/threonine kinases. CDC42BPG is an understudied kinase predicted to be downstream of CDC42 involved in cytoskeletal regulation based upon the functions of its two closest paralogs CDC42BPA/B (41). CDK19 is a member of the mediator complex controlling transcriptional activation (42). CDKL5 (also called STK9) function is poorly understood but is thought to be involved in regulation of ciliogenesis (43). Mutation of the CDKL5 gene results in CDKL5 deficiency disorder with CDKL5 playing a major role regulating neuronal survival during brain aging (44). The upregulation of protein kinases including RTKs was largely the result of increased transcription resulting from a rapid degradation of the super transcription factor c-MYC, which regulates expression of up to 15% of the human genome (28, 29, 35, 45). c-MYC is stabilized by phosphorylation at Ser62 and inhibition of the MEK-ERK pathway by selumetinib or trametinib resulted in loss of P-Ser62 and rapid degradation of c-MYC (28, 29). Turnover of c-MYC has been shown to regulate genomic reorganization and recruitment of transcriptional complexes to genomic loci including those regulating transcription of specific kinases (46).

In HER2+ amplified breast cancer cell lines, treatment with lapatinib induces an adaptive reprogramming of the kinome involving transcriptional upregulation of multiple tyrosine kinases including HER3, IGF1R, DDR1, MET, FGFRs and reactivation of HER2/HER3 signaling resulting in reactivation of AKT signaling, tumor cell survival, and proliferation (11, 47). RNAseq was then coupled with ChIPseq analysis to determine epigenetic changes regulating transcriptional reprogramming in HER2+ cell lines (47). Motif analysis of ChIPseq data that used antibodies to BRD4, a component of the mediator complex, which recruits RNA polymerase II transcription machinery MED1 and H3K27ac of lapatinib-responsive genomic regions, identified FOXA1 as a transcription factor mediating adaptive responses. Combination treatment with lapatinib and FOXA1 RNAi knockdown resulted in decreased proliferation and in dysregulation of enhancers remodeled in response to lapatinib including one known to regulate HER3 expression. Based on these studies, a window-of-opportunity trial examined adaptive changes in women with HER2+ breast cancer in response to current standard-of-care anti-HER2 therapies: trastuzumab, pertuzumab, trastuzumab + pertuzumab, and trastuzumab + lapatinib (ClinicalTrials.gov NCT01875666). Patients were given treatment for 7 days with biopsies taken before and after the 7-day treatment cycle for analysis by RNAseq (Fig. 1). Transcriptional analysis combined with MIB/MS proteomic analysis of the kinome of patient post/pretreatment biopsies demonstrated overlapping but distinct kinome reprogramming in all treatment arms as well as immune responses characteristic of HER2 antibody treatment (47). In addition, a subset of HER2+ patient tumors had stronger molecular responses exhibiting a significant reduction in expression of FOXA1, HER2, HER3, CDK1, and other proliferative kinases. FOXA1 served as a master transcription factor in the adaptive response in regulating HER3 expression in the HER2+ patient trial reinforcing the hypothesis that inhibiting FOXA1 transcriptional regulatory activity may prove effective as a novel therapy in combination with HER2 inhibition. Taken together, these unique window-of-opportunity trials suggest a predominantly transcriptional mechanism mediating adaptive kinome reprogramming and drug resistance resulting from epigenetic remodeling of the promoter and enhancer landscape.

## Adaptive RTK reprogramming in response to mutant KRAS(G12C) inhibition

RAS proteins (Kirsten rat sarcoma viral oncogene homolog [KRAS], Harvey rat sarcoma viral oncogene homolog, neuroblastoma rat sarcoma viral oncogene homolog) are GTPases that regulate kinase signaling pathways including the RAF-MEK-ERK pathway and are frequently activated by specific mutations in cancer. KRAS glycine 12 mutated to cysteine (KRAS[G12C]) is an activating KRAS mutant found in ∼12% of lung adenocarcinomas, ∼3% of colon cancers, and a lower frequency in pancreatic adenocarcinomas and other cancers. The cysteine mutation at this critical glycine 12 residue has allowed the development of highly selective irreversible inhibitors of KRAS(G12C) (48, 49). These inhibitors have shown promising results in phase I trials in a small number of patients with tumor arrest or tumor shrinkage (50, 51). However, like adaptive responses with kinase inhibitors in cell lines, mouse patient-derived xenograft (PDX) models, and patients, the response may be short-lived, and adaptive resistance develops (51, 52, 53, 54). Adaptive resistance frequently involves the expression of RTKs able to reactivate the RAF-MEK-ERK pathway *via* activation of wild-type KRAS, Harvey rat sarcoma viral oncogene homolog, or neuroblastoma rat sarcoma viral oncogene homolog, which are not inhibited by the cysteine-targeted KRAS inhibitors (53, 54, 55). The RTK-driven bypass of KRAS(G12C) inhibition may also involve PI3K-AKT pathway activation. Tumor cells may express more than one specific RTK making it difficult to use single kinase inhibitors in combination with the irreversible KRAS(G12C) inhibitor. Ryan *et al.* used a panel of lung, colon, and pancreatic tumor cell lines expressing KRAS(G12C) to demonstrate that the protein tyrosine phosphatase, SHP2 (PTPN11), was required for activation of the wild-type rat sarcoma viral homolog [RAS] proteins in the background of KRAS(G12C) inhibition (54). SHP2 is a tyrosine phosphatase required for RTK activation of RAS proteins (56, 57), and the combination of SHP2 and KRAS(G12C) inhibition was able to give a durable inhibition of ERK activity and growth arrest (54).

**Figure 2:**
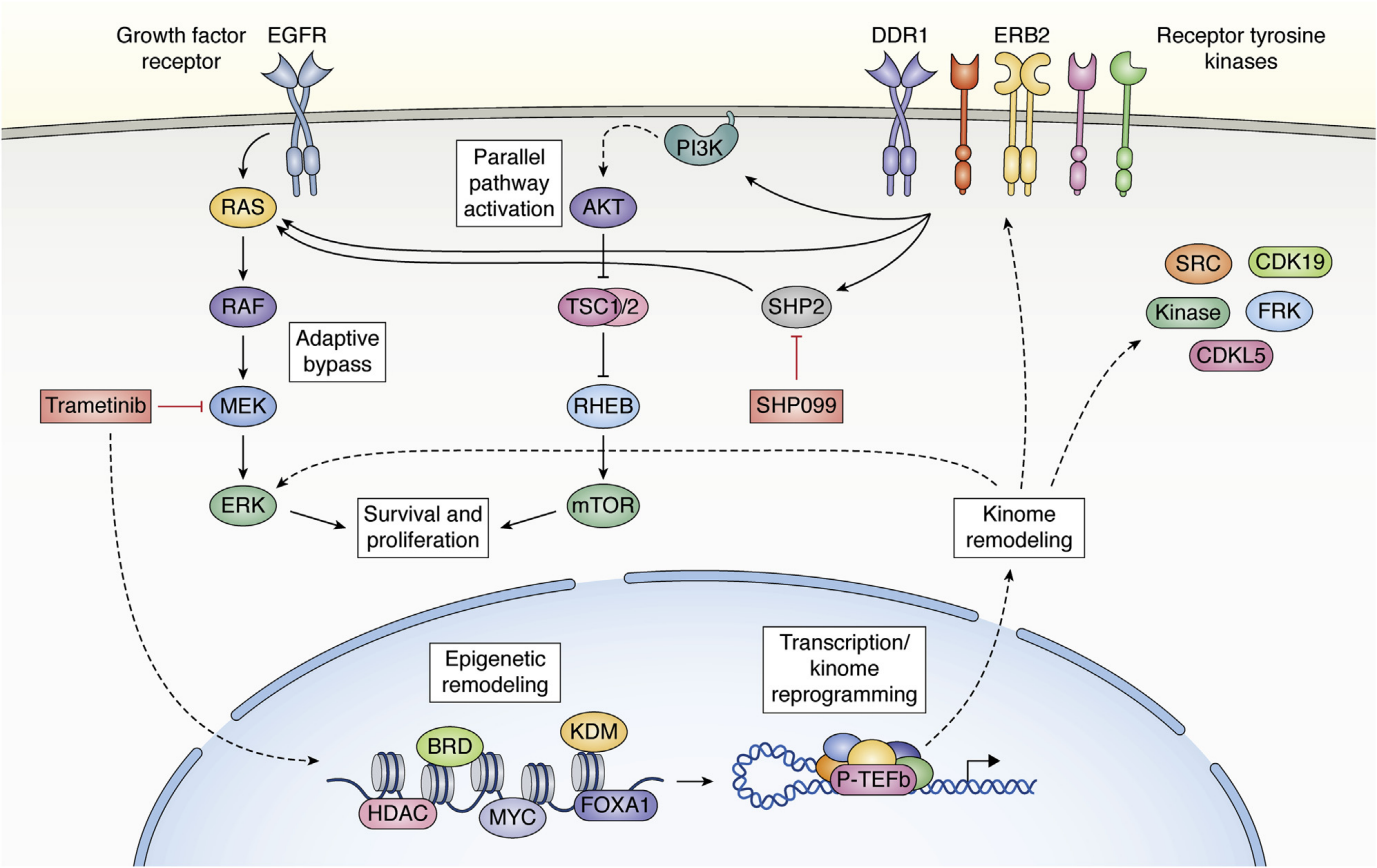
**Model of drug tolerance and adaptive resistance in response to targeted kinase inhibition.** Inhibition of a specific kinase (*e.g.*, trametinib inhibits MEK) drives remodeling of the epigenetic landscape through different transcription factors and epigenetic readers and writers depending on the inhibitor used and cellular context. Changes in the enhancer and promoter landscape drive transcriptional reprogramming of the kinome leading to upregulation of receptor tyrosine and other kinases. Kinome remodeling results in cell survival and proliferation by activating parallel proliferative pathways including the PI3K-AKT signaling pathway or through reactivation of the inhibited pathway (*e.g.*, MEK-ERK). Pathway reactivation occurs when activity of upstream kinases is increased such that targeted inhibition is overcome in a mechanism known as adaptive bypass. Activation of parallel and/or vertical pathways compensates for the inhibited target to allow for cell survival and proliferation in a mechanism termed adaptive resistance. The rational design of combination therapies targeting each step of the adaptive resistance pathway has shown promise in clinic trials and in preclinical models including combination of MEK or KRAS(G12C) inhibitors with an allosteric inhibitor (SHP099) of the tyrosine phosphatase SHP2. Blocking adaptive resistance pathways with such combinations will be critical to making targeted kinase inhibitors have more durable responses in patients.

The use of cotargeting SHP2 and KRAS(G12C) with selective inhibitors is a novel example of vertical pathway inhibition (Fig. 2) (55). Vertical pathway inhibition has been found useful, for example, in late stage, metastatic melanoma with the combination of a BRAF inhibitor and MEK inhibitor (58, 59, 60). Generally, the success of vertical pathway inhibition has involved targeted inhibitors of two or more kinases within the same pathway (58, 59, 60, 61). Resistance generally develops to vertical pathway inhibition by activation of parallel pathways that overcome single pathway inhibition or by acquired resistance with mutation of one of the targeted kinases (54). Use of a SHP2 inhibitor (SHP099) is somewhat different in that the protein tyrosine phosphatase is required for RAS activation by different RTKs and thus, may have a more durable inhibitory action when combined with KRAS(G12C) inhibition than targeting specific RTKs or other kinases in a pathway with selective inhibitors for each kinase. An obvious current limitation is the infrequent expression of the KRAS(G12C) mutant in different cancers that allows the use of an irreversible covalent cysteine reactive inhibitor.

Ahmed *et al.* studied the dependence of SHP2 in MEK-ERK pathway resistance in response to MEK inhibition in different tumor types including breast, colon, and thyroid cancers (62). It was found that SHP2 inhibitor (SHP099) was effective in combination with MEK inhibitor in the presence of wild-type RAS or RAS (G12X) mutants, but RAS (G13D) and (Q61X) mutants were resistant to SHP2 inhibition and overcame growth inhibition with the combination MEK and SHP2 inhibitors. It was also found that some tumors expressing a receptor tyrosine kinase FGFR receptor in the background of a BRAF (V600E) mutation were resistant to the SHP2 inhibitor. Cell lines were also identified that had very low SHP2 phospho(Y542), used as a surrogate marker for SHP2 activation, which were resistant to the small-molecule inhibitor SHP099. Given the diversity of RTK/TKs expressed in the adaptive reprogramming response in patient tumors, including upregulation of FGFR, it is possible there are additional kinases that activate the RAS-BRAF-MEK-ERK pathway independent of SHP2 that will be resistant to SHP2 inhibition. The RAS initiative may overcome this limitation with new mutant RAS selective inhibitors or RAS mutant selective protein degraders that would be effective in a vertical pathway with MEK inhibitors.

## Open chromatin states and enhancer remodeling drive adaptive kinome reprogramming in response to targeted kinase inhibitors

In breast cancer, a spectrum of interconvertible phenotypic and functional states has been linked to clinical drug resistance (26, 63, 64), which is consistent with circulating tumor cells not being committed to a single cell state (65, 66). Remodeling of open chromatin that alters functional gene transcription can regulate cell state switching that can give rise to drug-tolerant persister phenotypes (Fig. 2) (22, 67). The changes in epigenetic reprogramming involve genes in addition to protein kinases (26, 29). It is possible to block these open chromatin state transitions with BET bromodomain inhibitors (26) and suppress recruitment of BRD4, MED1, and CBP/p300 to newly formed enhancers and the transcriptional regulatory pTEFb complexes (29); thus, effectively blocking the persister phenotype.

A cell system we have used to address this question is the SUM229PE TNBC cell line. SUM229PE cells maintain two populations in culture: one epithelial and basal-like (SUM229PE EpCAM+ referred to as POS) and the second mesenchymal and claudin low-like (SUM229PE EpCAM-referred to as NEG) (68). Exome sequencing showed that POS and NEG SUM229PE cells have no unique nucleotide or copy number alterations (69). In addition, genome-wide DNA methylation sequencing showed no significant difference between the two populations that would give a differential transcriptional response to MEK-ERK inhibition with trametinib treatment. Jordan *et al.* performed an siRNA screen in the SUM229PE NEG cells showing that SMARCD3/BAF60C, a regulatory subunit of the SWI/SNF chromatin remodeling complex, was necessary to maintain the mesenchymal phenotype (70). Loss of Smarcd3/Baf60c expression converted SUM229PE NEG cells to the epithelial EpCAM+ (POS) phenotype, consistent with the POS and NEG phenotypes being controlled by differing chromatin states (69). FAIRE (Formaldehyde-Assisted Isolation of Regulatory Elements) sequencing showed differences in open chromatin accessibility between POS and NEG cells that correlated with histone H3 lysine 27 acetylation (H3K27ac) measured by ChIPseq. This is consistent with studies showing that H3K27ac is associated with relaxed chromatin and enriched at enhancers along with BRD4, MED1, and the transcriptional coactivator p300 (29, 71). Genes associated with epithelial or mesenchymal phenotypes were differentially expressed in POS and NEG cells, respectfully (69). For example, a subset of genes preferentially expressed in POS cells regulate cell–cell adhesion, a subset of genes expressed in NEG cells regulate binding to extracellular matrix. NEG cells express genes found in mesenchymal breast cancer such as the transcription factor ZEB1 and the collagen component COL6A2; POS cells express genes associated with basal breast cancers, such as the GRHL2 transcription factor, the cell adhesion protein cadherin (CDH1), and the cytoskeletal keratin KRT5. The POS and NEG SUM229PE subpopulations are differentially regulated by chromatin and transcriptional variation resulting in epithelial and mesenchymal differentiation states.

Trametinib treatment for 24 h of POS and NEG SUM229PE cells showed by RNAseq that ∼15% and ∼10% of their transcriptomes were altered greater than twofold, respectfully (69). Kinome transcriptome analysis of the POS and NEG cells showed kinases upregulated and downregulated in response to trametinib. Upregulated transcripts were enriched in tyrosine kinases (TKs) and TK-like kinases in both POS and NEG subpopulations, whereas downregulated transcripts were enriched in cell-cycle and mitotic checkpoint kinases. Kinases characteristic of EpCAM+, basal-like and EpCAM-, claudin-low TK transcriptional signatures were upregulated with trametinib treatment in parallel with EpCAM status in POS and NEG cells, respectively. PDGFRβ, a mesenchymal, claudin-low kinase, was selectively induced in NEG cells and FGFR2 increased in the epithelial, basal-like POS cells. The transcriptional response to trametinib showed a unique adaptive reprogramming in the two subpopulations of SUM229PE cells that demonstrates cells of similar genomic sequence but different in chromatin and epigenetic state have heterogeneous responses in the adaptive transcriptional response and expression of protein kinases. Similar expression differences in transcriptional signatures were seen in basal-like cell lines (HCC1806, SUM149PT EpCAM+, MDA-MB-468) *versus* claudin-low cell lines (SUM159PT, Hs578T, WHIM12) (29).

The change in transcriptional signatures in response to trametinib *versus* DMSO control was correlated in different basal-like and claudin-low cell lines with remodeling of enhancer regions using ChIPseq analysis of BRD4, H3K27ac, p300, and MED1 and promoter regions being defined by occupancy of H3K4me3 at the transcription start site (29). Trametinib treatment of SUM159PT claudin-low and HCC1806 basal-like cells nearly doubled their baseline enhancer number with 24 h trametinib treatment. Enhancer reprogramming could be observed within 1–4 h of trametinib treatment and was maximal by 48–72 h. Protein kinases transcriptionally upregulated with trametinib treatment of cells were driven by this adaptive enhancer formation. CRISPR deletion of the trametinib-induced DDR1 enhancer blocked transcriptional upregulation of DDR1 transcript and protein expression. Like the reversal of the adaptive drug-tolerant state, the newly formed enhancers and transcriptional upregulation were reversed by removal of trametinib from the growth media.

## Inhibition of adaptive kinome reprogramming

Tumor cells have interconvertible phenotypes with dynamic open chromatin states that allow differential gene transcription (26, 64). This phenotypic switching contributes to the heterogeneity of drug response and the drug-tolerant state that can lead to resistance. The dynamic state of open chromatin transcriptional control could result in the kinome adaptive response to vary in a tumor to a therapeutic drug. Furthermore, the off-target action of ATP competitive inhibitors, which is seen with almost all ATP-binding site kinase inhibitors, can contribute to the heterogeneity of adaptive kinome reprogramming. An example of the complexity of the heterogeneity of drug response is demonstrated by the study by Miao *et al.* (31). They employed a chemoproteomics approach to study the differences in kinome reprogramming in the M14 melanoma cell line between two FDA-approved BRAF inhibitors, Dabrafenib and Vemurafenib. Chemical structures and specificity profiles of these two BRAF(V600E) inhibitors are different (72). In M14 melanoma cells, the two inhibitors used as single agents targeting BRAF(V600E) differentially upregulated the expression of specific tyrosine kinases (31). Dabrafenib increased expression of DDR1, HER3, JAK1, and PDGFRβ, whereas Vemurafenib upregulated ABL1, EPHB4, FES, LYN, and TK1. The only commonly upregulated tyrosine kinase was PTK7. The differences in kinome reprogramming are a product of the off-target selectivity of the compounds, not their common inhibition of BRAF(V600E).

The dynamic phenotypic heterogeneity of tumor cells, selection of drug-resistant persister cells giving a drug tolerant state, and the on-target, off-target action of kinase inhibitors result in a complex cellular response that must be abrogated to arrest adaptive reprogramming of the kinome to make patient therapies durable. What this really means is that most single agent kinase inhibitor therapies will not have durable responses and as stated by Yaeger and Solit in reviewing adaptive responses to KRAS(G12C) inhibitors (55) “mechanistically informed combinations will be required to achieve durable responses in most patients.” Informed combination therapies are not easily envisioned for cancers whose adaptive response to kinase inhibitors (or RAS inhibitor) involves: (1) Loss of feedback inhibition of TK activation of signaling networks controlling tumor cell growth, survival, and migration; and (2) Chromatin remodeling and enhancer formation that drive significant transcriptional changes including expression of RTKs, other TKs, serine-threonine-directed kinases, and lipid kinases.

Dabrafenib and Vemurafenib both inhibit BRAF(V600E), but resistance develops with single agent Dabrafenib or Vemurafenib treatment. Both BRAF(V600E) inhibitors are approved for use in combination with a MEK inhibitor for treatment of advanced melanoma and demonstrate an example of a successful vertical pathway inhibitor combination (58, 59, 60). SHP2 inhibitors, as discussed above, are another example of vertical pathway inhibition of RTK activation of RAS proteins (54), but SHP2 inhibitors do not block the adaptive transcriptional activation that leads to a functional phenotypic state change of the tumor cell. For example, trametinib inhibition of the MEK-ERK pathway in many tumor cells leads to a rapid degradation of c-MYC and transcriptional reprogramming that in some tumor cells can be 15–20% of the transcriptome, effectively changing the tumor cell phenotype (28, 29).

A second strategy for combination therapies is to block the transcriptional response to specific kinase inhibitors targeting pathways that control proliferation and survival (*e.g.*, MEK-ERK; PI3K-AKT) (Fig. 2). We demonstrated the transcriptional adaptive response to trametinib inhibition of the MEK-ERK pathway in TNBC patient tumors and in HER2+ breast cancer patients in response to current standard-of-care anti-HER2 therapies (trastuzumab, pertuzumab, trastuzumab + pertuzumab, and trastuzumab + lapatinib) (47). MEK inhibition of TNBC cell lines and PDXs and lapatinib inhibition of HER2+ cell lines induced genome-wide remodeling of chromatin that included enhancer and superenhancer formation (29, 47). Enhancer/superenhancer formation involved the seeding of BRD4, MED1, H3K27 acetylation, and p300 that was shown to regulate transcriptional adaptation to MEK or HER2 inhibition in TNBC and HER2+ breast cancer models. BRD4 and CBP/p300 are also members of the P-TEFb transcriptional regulatory complex, which phosphorylates components of the RNA polymerase II complex to allow for productive mRNA elongation. Using selective bromodomain inhibitors for BRD4 or CBP/p300 arrested enhancer seeding and RTK upregulation in preclinical cell lines and mouse models of different tumor types (11, 29). BRD4 bromodomain inhibitors were also shown to reverse resistance induced by growing cells in the presence of a kinase inhibitor (*e.g.*, trametinib). Thus, pharmacologic targeting of proteins involved in enhancer remodeling and the transcriptional P-TEFb regulatory complex in conjunction with kinase pathway inhibition can induce a durable therapeutic response in preclinical cancer models. These results suggest that combining kinase and chromatin remodeling inhibitors may offer a durable treatment strategy to reduce adaptive resistance.

## Conclusions

Dynamic transcriptional responses to trametinib and current standard-of-care anti-HER2 therapies in TNBC and HER2+ breast cancers with 1 week of treatment showed the rapid change in interconvertible phenotypic states of the tumor cell in patients. The transcriptional response involves the expression of multiple RTKs in patients that in preclinical models are shown to contribute to overcoming growth inhibition and stimulate proliferation and survival. The dynamic transcriptional responses are seen in response to different kinase inhibitors in different tumor types (11, 29, 35, 37, 47, 69). Transcriptional reprogramming associated with epigenetic remodeling is also observed with other, nonkinase inhibitors and chemotherapies (73, 74, 75, 76, 77). This rapid transcriptional response leads to short-term adaptive resistance over weeks that can be reversed by washout of the inhibitor, but with long-term growth in the presence of the inhibitor mutations may be selected that result in an acquired stable resistance (22). In some cancers where there is a mutant kinase driving the proliferation of the tumor cell by activating the MAPK pathway, such as mutant EGFR in NSCLC or BRAF(V600E) melanoma, vertical pathway combination kinase inhibitors maybe successful therapies in some patients. However, cancers such as TNBC do not have such driver mutations in proliferation and survival pathways. Rather TNBC is characterized, for example, as having frequent elevation of MAPK signaling resulting from elevated expression of wild-type KRAS, BRAF, or increased copy number of different RTKs (78), and a vertical pathway combination therapy has not been defined for TNBC. Thus, an important goal for TNBC and other cancers that do not have a defined driver mutation is to determine the chromatin landscape of cells in response to drug treatment and how to pharmacologically block adaptive and acquired chromatin states so that the primary drug treatment has a durable tumor inhibitory response.

Combination therapies involving targeted kinase inhibitors and chromatin remodeling inhibitors or SHP2 inhibitors could block the drug-tolerant state and make kinase inhibitor therapies durable (Fig. 2). For example, in TNBC trametinib causes a strong initial growth arrest that is rapidly bypassed by a selective chromatin remodeling and transcriptional response in a c-MYC-dependent process involving upregulated expression of RTKs. Trametinib in combination with a SHP2 inhibitor or a BET bromodomain inhibitor could be tested for the ability of the combination to inhibit and restore sensitivity to drug treatment. ClinicalTrials.gov currently lists nine phase 1 or phase 2 trials with different SHP2 inhibitors alone or in combination with other cancer drugs including MEK and ERK inhibitors. BET bromodomain inhibitors are also being tested in patients alone and in combination in treating different cancers (ClinicalTrials.gov). A concern is the toxicity of such combinations especially at maximally tolerated doses. Both SHP2 inhibitors and chromatin remodeling inhibitors will likely alter the activity of both vertical and parallel signaling pathways such as MEK-ERK and PI3K-AKT regulated by many RTKs. For example, targeting parallel pathways such as MEK-ERK and PI3K-AKT has shown significant toxicity in clinical trials (79, 80, 81). The recent success in using low-dose combinations of kinase inhibitors to successfully treat pancreatic, NSCLC, breast, and colon cancer cell lines, PDX, and mouse tumor models (see (82) for a recent review) suggests that similar strategies may work with low-dose chromatin remodeling or SHP2 inhibitors in combination with low-dose kinase inhibitors (61, 83, 84). The limitation with SHP2 inhibitors will depend on the mutant RAS, RTK profile, and level of SHP2 expression of the tumor cell (62). Success in the preclinical studies showing significant improvement in the durability of kinase inhibitor therapy has already led to combination therapy clinical trials with SHP2 inhibitors. It will be interesting to see if similar clinical trials can be pursued with chromatin remodeling inhibitors. An additional approach is the control of timing intervals of therapy, so-called drug holidays (85, 86). Adaptive transcriptional reprogramming is often reversible so the “persister tumor cells” can become resensitized to therapy. Thus, an understanding in patients of appropriate timing intervals of treatment with intermediate drug holidays could make therapies more durable. It is likely that for different tumors, specific therapies will require some combination of these approaches to make targeted kinase and RAS inhibitors durable.

## Conflict of interest

The authors declare that they have no conflicts of interest with the contents of this article.
